# Employee Green Behavior in Hospitals: A Scoping Review of Definitions and Influencing Factors

**DOI:** 10.34172/ijhpm.9377

**Published:** 2026-07-14

**Authors:** Seung Eun Lee, Heejin Lim, Hyeyeon Shin, Sungwon Park, Jin Jun

**Affiliations:** ^1^College of Nursing, Yonsei University, Seoul, Republic of Korea.; ^2^College of Nursing, Ohio State University, Columbus, OH, USA.; ^3^School of Nursing, University of California, Los Angeles, CA, USA.

**Keywords:** Green Behavior, Health Personnel, Hospital, Pro-environmental Behavior, Sustainability

## Abstract

**Background:**

Employee green behavior (EGB) refers to environmentally responsible actions undertaken by employees in the workplace. Although interest in EGB is growing, hospitals represent a uniquely complex environment where clinical demands, operational constraints, and regulatory pressures may shape such behavior in distinct ways. To date, no review has synthesized literature on EGB within hospital settings. This scoping review synthesizes existing research to clarify how EGB has been conceptualized and studied in hospitals, identify factors associated with EGB, and inform future research and sustainability efforts in healthcare organizations.

**Methods:**

This scoping review followed the Joanna Briggs Institute (JBI) methodology and reported per Preferred Reporting Items for Systematic Reviews and Meta-Analysis Extension for Scoping Reviews (PRISMA-ScR) guidelines. Seven databases (PubMed, CINAHL, Embase, Web of Science, Scopus, APA PsycINFO, and Emerald) and grey literature were searched from inception to January 31, 2025. Studies examining EGB among hospital employees were included.

**Results:**

Twenty-three studies met the inclusion criteria, with most published after 2021. The majority employed quantitative survey designs and focused on nurses, although some included multidisciplinary staff. Considerable variation was observed in how EGB was defined and measured across studies. Factors reported in relation to EGB were organized into four levels: individual, team, leadership, and organizational. Qualitative findings highlighted barriers such as time constraints, insufficient training, limited knowledge, and lack of leadership support, as well as facilitators including individual motivation, environmental awareness, shared responsibility, and access to sustainability resources and education.

**Conclusion:**

EGB in hospital settings has been defined and examined inconsistently in the existing literature. This review proposes a working definition of EGB as employee actions in the workplace that minimize environmental harm and promote sustainability. The findings indicate that EGB in hospitals is influenced by multiple factors, and effective sustainability efforts in healthcare organizations require coordinated multilevel approaches.

**Registry Name and Number:**

The review protocol was registered with the Open Science Framework (osf.io/kd57r).

## Background

 Hospitals are essential institutions dedicated to protecting, preserving, and restoring human health,^1^ yet they are also among the most resource-intensive facilities in modern society. Owing to their continuous operations, energy-intensive technologies, high material throughput, and reliance on single-use medical supplies, hospitals contribute substantially to environmental degradation through energy consumption, waste generation, greenhouse gas emissions, water use, and chemical pollution.^2,3^ If the global healthcare sector were considered as a country, it would rank among the largest emitters of greenhouse gases worldwide.^4^ These environmental impacts pose not only operational and financial challenges for hospitals but also ethical concerns, given their core mission to safeguard human health.^4^

 Environmental degradation and climate change are increasingly recognized as major public health threats.^1^ Rising temperatures and environmental changes are associated with a range of health risks^5^ and place growing pressure on healthcare systems and hospital services.^6^ Together, these dynamics highlight a fundamental paradox: hospitals are expected to mitigate the health consequences of environmental change while simultaneously contributing to its underlying drivers.^7^

 Although technological innovation, infrastructure development, and policy interventions are essential components of hospital sustainability efforts, researchers increasingly recognize that such measures alone are insufficient.^8,9^ Organizational sustainability requires more than formal compliance or policy implementation, and it depends on the everyday behaviors of employees. Decades of research on organizational citizenship behavior theory suggest that employees’ voluntary, extra-role actions, such as helping coworkers, being conscientious, and proactively addressing problems, cultivate the social and operational conditions necessary for long-term success.^10,11^ These discretionary behaviors also bolster collaboration, enhance efficiency, and foster a resilient work climate. As such, the cumulative effects of employee green behavior (EGB)—also referred to in prior literature as environmentally responsible or pro-environmental behavior in the workplace—have emerged as a critical mechanism through which organizations can reduce their environmental footprint.^12,13^ These behaviors include routine individual actions, such as conserving energy or managing materials responsibly, as well as more proactive and collective efforts, such as participating in environmental initiatives or promoting environmental awareness among colleagues.^14^

 A growing body of organizational and management research has examined EGB and its antecedents, including individual attitudes and values, leadership support, team dynamics, and organizational culture.^13^ This literature has also linked EGB to positive outcomes such as improved environmental performance, enhanced employee well-being, and higher job satisfaction.^13^ However,most existing studies have been conducted in non-healthcare sectors, such as manufacturing and hospitality,^13,15^ or have examined healthcare alongside other industries rather than as a distinct organizational context. A large meta-analysis of EGB research identified only a small number of studies conducted in healthcare settings,^16^ and subsequent reviews similarly included very few hospital-focused studies.^13,15^

 Hospitals represent an organizational context characterized by strict clinical protocols, regulatory oversight, patient safety imperatives, and time-critical decision-making processes that prioritize clinical effectiveness and compliance.^17,18^ These features may constrain employees’ discretion and shape how green behavior is defined, prioritized, and enacted in daily practice. In hospital settings, staff are often required to navigate competing priorities between patient care, operational efficiency, and environmental sustainability.

 Despite the increasing number of empirical studies examining EGB in hospitals, the literature has not yet been comprehensively mapped or synthesized. As a result, it remains unclear how EGB has been defined, measured, and examined across different levels of the hospital context. To date, no review has examined how EGB has been conceptualized and studied in hospital settings. This scoping review, therefore, aims to synthesize existing research on EGB in hospitals, clarify how EGB has been defined and studied, and map factors associated with EGB across multiple levels of the organizational context to inform future research and sustainability efforts in healthcare organizations.

## Review Questions

 This scoping review addresses the following research questions: (1) How has EGB been conceptualized and operationalized in hospital-based literature? and (2) What individual, team, leadership, and organizational factors have been reported as influencing EGB in hospital settings?

## Methods

 This scoping review followed the Joanna Briggs Institute (JBI) methodology for scoping reviews^19^ and is reported in accordance with the Preferred Reporting Items for Systematic Reviews and Meta-Analysis Extension for Scoping Reviews (PRISMA-ScR).^20^ A scoping review approach was selected to map the existing literature on EGB within hospital settings. This method is particularly well suited for examining an emerging and heterogeneous body of research, as it enables comprehensive mapping of key concepts, theoretical frameworks, and influential factors, rather than evaluation of effect sizes or causal relationships.^21^

###  Protocol and Registration

 The research objectives, questions, and methodological framework, including the eligibility criteria, search strategy, study selection, and approach to data analysis, were pre-specified in a registered protocol available on the Open Science Framework (osf.io/kd57r) and described in a peer-reviewed publication.^22^

###  Eligibility Criteria

 Eligibility criteria were established using the Population—Concept—Context framework.^19^ Studies were included if they focused on hospital employees of any professional background. With regard to the concept, studies examining EGB or related workplace pro-environmental behaviors were included, whereas studies focusing on environmentally related behaviors outside the workplace (eg, household pro-environmental behavior or sustainable consumer behavior) were excluded. In terms of context, only studies conducted in hospital settings were considered, as organizational context can influence employees’ engagement in green behavior.^13^ Studies conducted in other healthcare settings, such as nursing homes, were excluded.

 All empirical studies published in English were eligible for inclusion, regardless of publication year or study design (quantitative, qualitative, or mixed methods). Studies primarily evaluating the effectiveness of specific interventions (eg, educational programs on healthcare waste management) were excluded, as the objective of this review was to synthesize determinants and conceptualizations of EGB rather than intervention outcomes. Studies lacking sufficient methodological or results information (eg, protocols, conference abstracts) were excluded due to limited interpretability. Studies with unresolved internal inconsistencies between reported results (eg, discrepancies between text and tables) were also excluded; attempts to contact authors for clarification were unsuccessful.

###  Search Strategy

 The search strategy was developed in accordance with the JBI methodology^19^ using the Population (hospital workers)—Concept (EGB)—Context (hospital settings) framework. Search strings combined controlled vocabulary (eg, MeSH terms) and free-text keywords. The full search strategies are provided in the supplemental material (Table S1 in Supplementary file 1).

 A three-step search process was conducted with the assistance of a health sciences librarian. First, seven electronic databases (PubMed, Cumulative Index to Nursing and Allied Health Literature, Embase, Web of Science, Scopus, APA PsycINFO, and Emerald journals) were searched for peer-reviewed literature. Second, the reference lists of included studies were screened to identify additional relevant articles. Third, ProQuest Dissertations & Theses Global and Google Scholar were searched to identify relevant grey literature.

 The original review protocol^22^ included plans to search additional sources, such as the National Library of Medicine Bookshelf and websites of international organizations (eg, the United Nations, World Health Organization [WHO], and Intergovernmental Panel on Climate Change). However, these sources were excluded because they consisted primarily of policy documents or guidance reports rather than studies presenting original empirical data on EGB in hospital settings and therefore did not meet the inclusion criteria. The search included all studies published up to January 31, 2025, with no restriction on publication year.

###  Study Selection

 All retrieved records were imported to EndNote (version 20.6), and duplicates were removed. Two reviewers independently screened titles and abstracts, followed by full-text screening of potentially eligible studies. Disagreements were resolved through discussion with an additional third reviewer.

###  Data Extraction and Synthesis

 Data extracted from included studies comprised bibliographic information (title, first author, year of publication, country), study characteristics (design, sample, setting), theoretical frameworks or conceptual models, definitions or descriptions of EGB, and key findings. These data were organized and summarized in tabular form. Two reviewers independently extracted data, and a third reviewer cross-checked the extracted information for completeness and accuracy. Discrepancies were resolved through team discussion.

 To synthesize the quantitative findings, we used the integrative multilevel model proposed by Zacher et al^13^ as an organizing framework to categorize factors reported in the literature. Qualitative findings, including data from qualitative studies and open-ended responses in quantitative studies, were synthesized descriptively and organized into categories of facilitators and barriers. Consistent with the objectives of a scoping review, a formal risk-of-bias assessment was not conducted. Instead, key methodological characteristics of included studies (eg, study design and data collection methods) were extracted to inform interpretation of the evidence.

## Results

###  Search Outcomes

 The screening process of this scoping review is illustrated in the PRISMA-ScR flowchart diagram (Figure S1 in Supplementary file 2). The database search identified 6344 records, with an additional three records identified through ancestry searching. After removal of duplicates, 3005 records remained for title and abstract screening. Following full-text assessment, 23 studies met the inclusion criteria and were included in this scoping review.

###  Study Characteristics

 The 23 included studies varied in publication type, geographic distribution, research design, theoretical framework, and participant characteristics. Of these studies, 22 were peer-reviewed journal articles and one was a master’s thesis. Most studies (n = 19) were published in or after 2021, reflecting growing interest in environmental sustainability in healthcare in recent years (See Figure 1). The studies were conducted across multiple regions, with the largest proportion conducted in Asian countries (n = 11), followed by European countries (n = 6). Pakistan accounted for the highest number of studies (n = 5), followed by Brazil (n = 3), and China, Australia, and the Netherlands (n = 2 each). Several studies conducted in Pakistan and Brazil appeared to draw on overlapping data sources or institutional samples, raising the possibility of duplicated participant cohorts. As study-level identifiers were not consistently reported, overlap in the participant cohort could not be confirmed.

**Figure 1:**
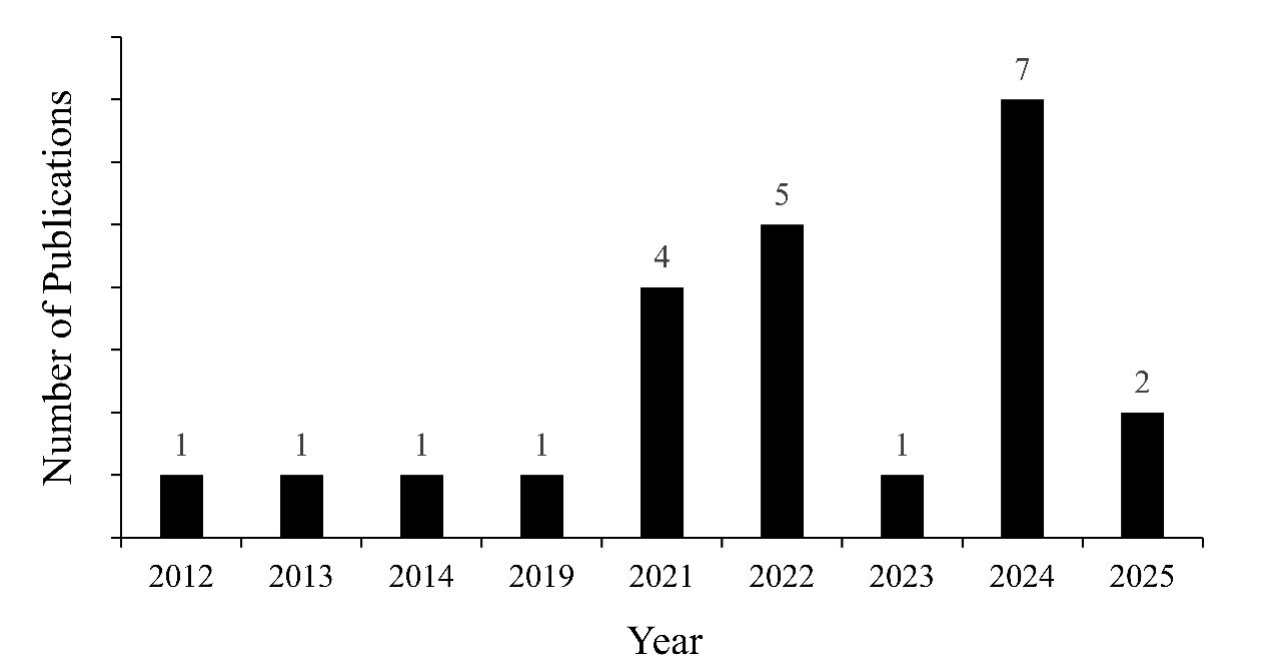
Number of Included Studies by Publication Year. Studies published up to January 31, 2025 were included without restriction on publication year.

 Regarding study design, quantitative survey-based methods were the most frequently used (n = 15), followed by qualitative design (n = 6), and mixed-method approaches (n = 2). Among quantitative studies, cross-sectional survey designs were most common (n = 10). Across these studies, a wide range of theoretical frameworks were employed. The most frequently used frameworks were the Theory of Planned Behavior^23^ and Social Learning Theory,^24^ followed by the Conservation of Resources Theory,^25^ Complexity Theory,^26^ and various organizational behavior models (See Table 1). Sample size ranged from 10 to 4552 participants. Demographic characteristics were not consistently reported across studies. Where age information was provided, most participants were in their 20s to 40s. Nurses were the most frequently studied professional group, followed by physicians, technicians, and administrative staff. Some studies focused on a single occupational group,^27-31^ whereas others involved multidisciplinary samples.^9,32-38^

**Table 1:** Overview of Study Designs, Theoretical Frameworks, and Sample Characteristics

**Authors **	**Year**	**Country**	**Aim**	**Study Design**	**Theoretical Framework**	**Sample**		
						**Number (F/M)**	**Age**	**Occupation**
Deng et al^8^	2022	Pakistan	To explain employees’ pro-environmental behavior from the standpoint of corporate social responsibility To test the mediating role of environmentally specific transformational leadership in influencing pro-environmental behavior	Time-lagged survey	SLT	293 (122/171)	22 – 40a	N/R
de Reeder^32^	2022	The Netherlands	To examine how patient safety culture affects EGB	Mixed method	TPB	80	25 – ≥65	Multiple roles
da Silva Diaz et al^33^	2013	Brazil	To discover the vision of individuals working in the emergency room of a teaching hospital on the management of hospital waste	Qualitative	N/R	17	N/R	Multiple roles
El-Sayed et al^27^	2024	Egypt	To examine the relationship between organizational agility and nurses' green work behavior and to assess how climate activism mediates this relationship	Cross-sectional	TPB, EMT, Green five taxonomy	781 (460/321)	32.76 ± 9.63	Nurse
Ferreira et al^39^	2024	Brazil	To unveil the meanings that nursing professionals attribute to practices related to waste management	Qualitative	Complexity theory	30	N/R	Nurse, nursing technician
Ferreira et al^40^	2024	Brazil	To understand the meanings revealed by nurses about sustainable care in its connections with the management of healthcare waste	Qualitative	Complexity theory	32	N/R	Nurse, nursing technician
Guihenneuc et al^34^	2024	France	To evaluate the knowledge, behaviors, practices, and expectations of health workers regarding climate change and environmental sustainability	Cross-sectional	N/R	4552 (3518/979)	≤29 – ≥60	Multiple roles
Hasan et al^41^	2024	Pakistan	To examine how eco-centric leadership impacts green knowledge-sharing attitudes among employees To investigate whether green climate mediates the relationship between eco-centric leadership and green knowledge sharing To explore whether green consciousness moderates the relationship between green knowledge sharing and the pro-environmental behavior of employees	Time-lagged survey	TPB, COR theory, SLT	251	N/R	Cleaner, sweeper
Li et al^28^	2021	China	To explore the relationship between green behavior intentions and green behavior and analyze the moderating role of ethical leadership in this relationship	Cross-sectional	N/R	489 (438/51)	26 – ≥31	Nurse
Liu et al^42^	2024	China	To develop a model to investigate whether organizational human resource management factors in the healthcare sector can induce employee voluntary green behavior	Time-lagged survey	SIP theory	223 (119/104)	43.41 ± 5.74	N/R
Mandal and Pal^43^	2025	India	To investigate how environment-specific servant leadership, via employee green value, promotes green organizational citizenship behavior	Cross-sectional	COR theory	333 (176/157)	18 – ≥40	N/R
McGain et al^29^	2012	Australia, New Zealand, England	To assess the views of anesthesiologists regarding recycling practices in operating rooms	Cross-sectional	N/R	780 (276/504)	≤45/>45	Anes.
Molnár et al^44^	2021	Pakistan	To investigate the interplay between corporate social responsibility at the employee level, quality of work life, ethical leadership, and employee pro-environmental behavior	Time-lagged survey	SLT, MEC theory, Theory of norm reciprocity	489 (256/233)	18 – ≥41	N/R
Peng et al^45^	2022	Pakistan	To improve the energy-specific pro-environmental behavior of hospital employees in an environmentally specific servant leadership framework	Time-lagged survey	SLT	316 (117/199)	22 – ≥41	N/R
Pinzone et al^35^	2019	Italy	To narrow the gaps by theorizing and empirically testing the effects of ‘green’ training on both employees’ organizational citizenship behaviors toward the environment performed by employees and job satisfaction	Cross-sectional	AMO theory, Goal setting theory	260 (211/49)	F: 46.2 M: 50.7	Multiple roles
Singleton et al^46^	2021	Australia, UK	To investigate factors affecting engagement with workplace pro-environmental behaviors in hospital pharmacists and pharmacy technicians To determine if legislated carbon reduction targets in the UK influenced workplace pro-environmental behaviors in the UK compared with Australia which does not have legislated carbon reduction targets	Mixed method	TPB, VBN theory, SCT, LOC theory, SDT, Reasonable person model, Goal framing theory	106 (70/36)	N/R	Pharmacist pharmacy technician
Sürme et al^30^	2025	Turkey	To explore the perspectives of nurses working in surgical intensive care units regarding responsible medical waste management, energy and medication consumption	Qualitative	N/R	23(17/6)	29.17 ± 6.26	Nurse
Tsai and Tan^36^	2022	Taiwan	To apply the theory of planned behavior and ethical leadership to determine the factors influencing the adoption of environmental protection behaviors	Cross-sectional	TPB	202 (117/199)	21 – ≥61	Multiple roles
van Nieuwenhuizen et al^47^	2024	Netherlands	To assess the various factors that influence environmentally sustainable behavior in gynecological surgery and examine the differences between gynecologists and residents	Qualitative	COM-B & BCW, Desmond framework	10	N/R	Gyn., resident
Vogt and Nunes^37^	2014	Germany	To investigate the personal and organizational factors influencing recycling behavior among hospital staff, mainly nurses	Cross-sectional	Work motivation theory	616 (487/129)	<20 – ≥60	Multiple roles
Widianto et al^38^	2021	Indonesia	To identify the influence of attitude, subjective norm, and behavioral control in its relation to awareness and the environmentally friendly behavior of the pharmacy staff	Cross-sectional	TPB	173	N/R	Multiple roles
Xu et al^9^	2022	Pakistan	To explore the factors that influence employees’ pro-environmental behavior in an organizational setting, especially their energy consumption behavior	Cross-sectional	SLT	441 (185/256)	18 – ≥41	Multiple roles
Zaw et al^31^	2023	Singapore	To analyze barriers and facilitators to green practices among anesthesiologists by understanding capability, opportunity, and motivation sources of behavior To understand the capability, opportunity, and motivational factors that influence green behavior will inform the design of effective interventions to promote environmentally sustainable practices in the operating room	Qualitative	COM-B & BCW	23 (14/9)	N/R	Anes.

Abbreviations: AMO, ability-motivation-opportunity; Anes.,anesthesiologist; COM-B & BCW, capability, opportunity, motivation, and behavior & behavior change wheel; COR, conservation of resources; EGB, employee green behavior; EMT, ecological modernization theory; F, female; Gyn., gynecologist; LOC, locus of control; M, male; MEC, means-end chain; N/R, not reported; SCT, social cognitive theory; SDT, self-determination theory; SIP, social information processing; SLT, social learning theory; TPB, theory of planned behavior; VBN, value-belief-norm.

###  Terminology, Conceptual Definitions, and Measurement Approaches

 The included studies used a range of terminologies, definitions, and measurement approaches to describe EGB in hospital settings. Although terminology and operationalization differed across studies, most addressed behaviors related to environmental sustainability within the workplace. The following subsections detail how terminology, conceptual definitions, and measurement strategies were applied (See Table 2).

**Table 2:** Overview of Terminologies, Definitions, and Measurement of Employee Green Behavior

**Authors**	**Year**	**Terminology Used**	**Definition/Description**	**Measure**
Deng et al^8^	2022	Pro-environmental behavior	An individual’s adaptive behavior to avoid actions that can harm nature and the built environment	Organizational citizenship behavior for the environment scale^48^
El-Sayed et al^27^	2024	EGB	Individual actions that align with sustainable development goals	EGB scale^49^
Guihenneuc et al^34^	2024	Pro-environmental behavior	Not explicitly defined	Author-developed scale
Hasan et al^41^	2024	Pro-environmental behavior	Initiative taken to enhance the environmental sustainability of the employer’s organization that is not mandated by formal environmental management policies and procedures	Not reported
Li et al^28^	2021	Green behavior	An element of environment-friendly behavior within organizational citizenship behavior and considered an essential component of organizational sustainable development that reflects the sense of responsibility of citizens at the ethical level	EGB scale^50^
Liu et al^42^	2024	Voluntary green behavior	Individuals voluntarily taking actions to benefit the environment beyond their organization’s formal awards	Employees’ pro-environmental behavior scale^51^
Mandal and Pal^43^	2025	Green organizational citizenship behavior	A specialized form of organizational citizenship behavior, emphasizing actions that promote environmental sustainability alongside the smooth functioning of the organization	Organizational citizenship behavior for the environment scale^52^
McGain et al^29^	2012	Recycling behavior	Not explicitly defined	Author-developed scale
Molnár et al^44^	2021	Employee pro-environmental behavior	Followers’ corporate social responsibility related to ethical behavior which is conducted in a discretionary or extra-role	Daily task-related pro-environmental behavior scale^53^
Peng et al^45^	2022	Energy-related pro-environmental behavior	A set of individual actions that influence energy consumption and production	Pro-environmental behavior scale^54^
Pinzone et al^35^	2019	Organizational citizenship behaviors toward the environment	Individual and discretionary social behaviors not explicitly recognized by the formal reward system and contributing to improve the effectiveness of environmental management of organizations Voluntary behaviors not specified in official job descriptions that, through the combined efforts of individual employees, help to make the organization and/or society more sustainable	Organizational citizenship behavior for the environment scale^52^
Tsai and Tan^36^	2022	Environmental protection behavior	Behaviors ranging from recycling behaviors, pollution reduction to energy saving according to the theory of planned behavior	Author-developed scale
Vogt and Nunes^37^	2014	Pro-environmental behavior	Consciously seeking to minimize the negative impacts of one’s actions on the natural and man-made environment through minimization of resource and energy consumption, use of non-toxic substances and reduced waste production	Author-developed scale
Widianto et al^38^	2021	Green behavior	Not explicitly defined	Not reported
Xu et al^9^	2022	Pro-environmental behavior	Acting in ways that produce minimum harm to the environment	Pro-environmental behavior scale^54^
de Reeder^32^	2022	EGB	Behavior conforming to organizational policies and procedures and choosing responsible alternatives in methods of work Behavior that goes beyond organizational expectations and involves personal initiative that exceeds organizational expectations	Employee task performance scale^55^; Organizational citizenship behavior for the environment^52^ Semi-structured interview, Observation
Singleton et al^46^	2021	Workplace pro-environmental behaviors	Actions and behaviors that employees consciously engage in to minimize the negative impact of the organization’s activities on the natural and built world	Semi-structured interview
da Silva Diaz et al^33^	2013	Vision on healthcare waste management	Not explicitly defined	Semi-structured interview
Ferreira et al^39^	2024	Healthcare waste management practices	Not explicitly defined	Semi-structured interview
Ferreira et al^40^	2024	Sustainable care meaning	Not explicitly defined	Semi-structured interview
Sürme et al^30^	2025	Sustainable waste/energy/medication management	Not explicitly defined	Semi-structured interview
van Nieuwenhuizen et al^47^	2024	Environmentally sustainable behavior	Not explicitly defined	Semi-structured interview
Zaw et al^31^	2023	Green behavior	Actions and behaviors that minimize the environmental impact of anesthetic procedures, such as the reduction of volatile anesthetic use and recycling of materials in the operating room	Semi-structured interview

Abbreviation: EGB, employee green behavior.

####  Terminology

 The most commonly used terms across the included studies were “pro-environmental behavior,”^8,9,34,37,41^ “green behavior,”^28,31,38^ and “EGB.”^27,32^ These terms were often used interchangeably to describe environmentally supportive actions taken by healthcare workers. Additionally, some studies employed more specific terminology such as “green organizational citizenship behavior,”^43^ “voluntary green behavior,”^42^ and “environmentally sustainable behavior.”^47^

####  Conceptual Definitions

 Conceptual definitions or descriptions of EGB varied across the included studies. Some studies characterized EGB as discretionary or voluntary actions that extend beyond formal job requirements.^28,32,35,41,43,44^ Others described it as ethically motivated behavior^28,44^ or as stemming from personal responsibility and initiative,^8,9,37,42^ while some highlighted alignment with organizational goals.^27,32,35,41,43,44^ A few studies focused on specific domains, such as energy-related pro-environmental behavior^45^ or recycling.^29^ Eight studies did not provide clear conceptual definitions or descriptions of green behavior.^29,30,33,34,38-40,47^

####  Measurement Approaches

 Most studies assessed EGB using structured survey measures. Several studies utilized previously validated instruments, such as the Organizational Citizenship Behavior for the Environment Scale^52^ and the Pro-environmental Behavior Scale.^54^ Other studies employed author-developed survey instruments tailored to the distinct environmental practices of healthcare settings.^29,34,36,37^ Qualitative studies primarily used semi-structured interviews to explore environmentally related behaviors in hospital contexts.

####  Variation in Terminology, Definitions, and Measurement

 Several inconsistencies were observed across the included studies regarding terminology, conceptual definitions, and measurement tools. Terms such as “green behavior,” “pro-environmental behavior,” and “EGB” were often used interchangeably, yet defined in varying ways. For example, some studies defined pro-environmental behavior as discretionary workplace actions,^41^ while others described it in ecological or technical terms.^8,9,37^ Similarly, the same scales, such as the Organizational Citizenship Behavior for the Environment Scale,^52^ were used across studies despite differing underlying definitions.^35,43^ These observed inconsistencies reflect a lack of standardization in terminology and construct definition in hospital-based green behavior research.

###  Factors Associated with Employee Green Behavior Based on Quantitative Findings

 This section synthesizes quantitative evidence on factors reported to influence green behavior in hospital settings. Because most of the included studies employed cross-sectional designs, the findings should be interpreted as associations rather than causal relationships. For the purpose of synthesis, the identified factors were organized into four categories: individual, team, leadership, and organizational. An overview of the identified variables and their reported associations is provided in Table 3.

**Table 3:** Factors Associated With Employee Green Behavior From Quantitative Findings

**Category**	**Sub-category**	**Variables**	**Type of Effect**	**Relationship**	**Key Findings Summary**
Individual	Demographic	Age	Direct	Positive	Older age was associated with higher proactive green behavior^32^ and green work behavior^27^
			Direct	Not significant	Age is not significantly related to organizational citizenship behavior for the environment^35^ and pro-environmental behavior^37^
		Educational level	Direct	Group difference	Employees with doctoral degrees showed higher green work behavior than those with diploma degrees^27^
		Gender	Direct	Group difference	Males showed higher pro-environmental behavior than females^34^
			Direct	Not significant	Gender is not significantly related to organizational citizenship behavior for the environment^35^ and pro-environmental behavior^37^
		Job tenure	Direct	Positive	Longer tenure was associated with more green work behavior,^27^ pro-environmental behavior,^34^ and organizational citizenship behavior for the environment^35^
	Job-related	Direct interaction with patients	Direct	Group difference	Employees directly interacting with patients showed less proactive green behavior than those who did not^32^
		Work shift	Direct	Group difference	Night shift nurses showed higher green work behavior than morning and evening shift nurses^27^
	Psychological/motivational/value-related	Altruistic attribution	Direct, moderation	Positive	Altruistic attribution was positively associated with voluntary green behavior and strengthened the indirect effect of socially responsible human resource management on voluntary green behavior through green passion^42^
		Altruistic value	Moderation	Positive	Altruistic value strengthened the relationship between corporate social responsibility and pro-environmental behavior,^8^ and the relationship between corporate social responsibility and organizational green practice, which influenced pro-environmental behavior^9^
		Climate activism	Direct, mediation	Positive	Climate activism positively influenced green work behavior and mediated the positive relationship between organizational agility and green work behavior^27^
		Green behavior intention	Direct	Positive	Green behavior intention positively predicted green behavior,^28^ environmentally friendly behavior,^38^ and environmental protection behavior^36^
		Green consciousness	Direct, moderation	Positive, negative	Green consciousness positively predicted pro-environmental behavior and negatively moderated the relationship between green knowledge sharing and pro-environmental behavior^41^
		Green goal difficulty	Direct, mediation	Positive	Green goal difficulty was positively associated with organizational citizenship behavior for the environment and mediated the relationship between green training and organizational citizenship behavior for the environment^35^
		Green passion	Direct, mediation	Positive	Green passion was positively related to voluntary green behavior and mediated the effect of socially responsible human resource management on voluntary green behavior^42^
		Green self-efficacy	Direct, mediation	Positive	Green self-efficacy positively predicted energy-related pro-environmental behavior and mediated the relationship between environment-specific servant leadership and energy-related pro-environmental behavior^45^
		Green value	Direct, mediation	Positive	Green value positively predicts green organizational citizenship behavior and partially mediates the relationship between environmental-specific servant leadership and green organizational citizenship behavior^43^
		Positive affect	Direct	Not significant	Positive affect was not significantly associated with organizational citizenship behavior for the environment^35^
		Pro-environmental attitude	Direct	Positive, negative	Pro-environmental attitude had a positive association with proactive green behavior and a negative association with task-related green behavior^32^
		Quality of work life	Moderation	Positive	Quality of work life strengthened the indirect effect of corporate social responsibility through ethical leadership on pro-environmental behavior^44^
		Self-serving attribution	Direct, moderation	Negative	Self-serving attribution negatively predicted voluntary green behavior and weakened the indirect effect of socially responsible human resource management on voluntary green behavior through green passion^42^
	Exposure-related	Previous attendance of workshops	Direct	Group difference	Employees who had previously attended climate change workshops showed higher green work behavior than those who had not^27^
Team		Green knowledge sharing	Direct, mediation	Positive	Green knowledge sharing was positively associated with pro-environmental behavior and mediated the relationships between eco-centric leadership, green climate, and pro-environmental behavior^41^
		Green teams	Direct	Group difference	Employees working in the department with green teams showed higher proactive green behavior than those without^32^
Leadership		Eco-centric leadership	Direct	Positive	Eco-centric leadership positively predicted pro-environmental behavior^41^
		Environmental-specific servant leadership	Direct	Positive	Environmental-specific servant leadership directly promoted green organizational citizenship behavior^43^ and energy-related pro-environmental behavior^45^
		Environmental-specific transformational leadership	Direct, mediation	Positive	Environmental-specific transformational leadership positively predicted pro-environmental behavior and mediated the relationship between corporate social responsibility and pro-environmental behavior^8^
		Ethical leadership	Direct, mediation, moderation	Positive	Ethical leadership positively predicted green behavior^28^ and pro-environmental behavior,^44^ partially mediated the relationship between corporate social responsibility and pro-environmental behavior,^44^ and strengthened the relationship between green behavior intention and green behavior^28^
Organizational	Structural	Corporate social responsibility	Direct	Positive	Corporate social responsibility positively predicted pro-environmental behavior^8,9,44^
		Eco-certified hospitals	Direct	Group difference	Employees working in eco-certified hospitals reported higher satisfaction with waste management systems and fewer perceived problems with waste handling compared to those in non-certified hospitals^37^
		Green training	Direct	Positive	Green training positively predicted organizational citizenship behavior for the environment^35^
		Organizational green practice	Mediation	Positive	Organizational green practice mediated the relationship between corporate social responsibility and pro-environmental behavior^9^
		Organizational orientation regarding sustainability	Direct	Positive	Organizational orientation regarding sustainability was positively associated with task-related green behavior^32^
		Socially responsible human resource management	Direct	Positive	Socially responsible human resource management directly predicted voluntary green behavior^42^
		Sustainable development steering committee	Direct	Group difference	The presence of a sustainable development steering committee was positively associated with the initiation of environmental sustainability actions^34^
	Cultural	Green work climate	Direct	Positive	Green work climate was positively associated with proactive green behavior^32^ and pro-environmental behavior^41^
		Organizational agility	Direct	Positive	Organizational agility positively predicted green work behavior^27^
		Patient safety culture	Direct	Positive	Patient safety culture positively predicted proactive green behavior^32^
		Perceived organizational environmental support	Direct, mediation	Positive	Perceived organizational environmental support was positively associated with energy-related pro-environmental behavior and mediated the relationship between environment-specific servant leadership and energy-related pro-environmental behavior^45^
			Direct	Negative	Perceived organizational environmental support was negatively associated with task-related green behavior^32^

####  Individual-level Factors

 Individual-level influencing factors include demographic, job-related, psychological/motivational/value-related, and exposure-related variables. Findings on demographic predictors such as age and gender were mixed. Some studies found that older or experienced employees were more likely to engage in green behaviors,^27,32^ while others reported no significant associations with age.^35,37^ Similarly, gender effects varied across studies: one study found higher pro-environmental behavior among male employees,^34^ whereas others found no significant differences by gender.^35,37^ Higher levels of education were positively associated with green behavior in one study.^27^ Job-related factors such as direct interaction with patients^32^ and work shift^27^ showed group differences, with non-clinical and night-shift staff reporting higher levels of environmentally sustainable behavior.

 Several psychological, motivational, and value-related variables emerged as relevant factors influencing green behavior. For example, green goal difficulty,^35^ green passion,^42^ and green self-efficacy^45^ were directly associated with green behavior and also examined as mediators linking leadership or organizational factors to behavioral outcomes. Similarly, green values^43^ and climate activism^27^ were associated with green behavior and were discussed as potential mediators. Some psychological factors were also examined as moderators. For instance, green consciousness was positively associated with pro-environmental behavior, but was reported to attenuate the association between knowledge sharing and behavior.^41^ In contrast, altruistic value and altruistic attribution were found to strengthen the association between corporate social responsibility^8,9^ or socially responsible human resources management^42^ on green behavior, showing the conditional nature of these psychological influences.

####  Team-Level Factors

 At the team level, green knowledge sharing was associated with green behavior and was also identified as a mediator in the relationship between leadership, green climate, and pro-environmental behavior.^41^ Additionally, the presence of designated green teams was associated with significantly higher levels of engagement in proactive green behaviors.^32^

####  Leadership-Level Factors 

 Various leadership styles, including eco-centric,^41^ environment-specific servant,^43,45^ environmental-specific transformational leadership,^8^ and ethical leadership,^28,44^ were associated with higher levels of green behavior. In some studies, these leadership variables were also found to mediate the relationship between organizational factors and employee behavioral outcomes.^8,44^

####  Organizational-Level Factors

 Organizational-level influencing determinants included both structural and cultural factors. Corporate social responsibility was one of the most frequently examined organizational factors and was associated with green behavior both directly and indirectly through mediators such as organizational green practices^9^ and environmental-specific transformational leadership.^8^ Other structural supports included green training,^35^ socially responsible human resource management,^42^ and organizational orientation regarding sustainability.^32^ The presence of eco-certified hospitals^37^ and sustainable development steering committees^34^ was also associated with higher levels of EGB. Cultural factors such as green work climate,^32,41^ organizational agility,^27^ and a strong patient safety culture^32^ were positively related to EGB.

###  Barriers and Facilitators for Employee Green Behavior Based on Qualitative Findings

 The qualitative findings on key barriers and facilitators influencing EGB among hospital employees are summarized in Table 4. For the purpose of synthesis, the identified barriers and facilitators were organized into four categories: individual, team, leadership, and organizational.

**Table 4:** Barriers and Facilitators for Employee Green Behavior From Qualitative Findings

**Category**	**Barriers**	**Facilitators**
Individual	Limited or fragmented knowledge of how to execute waste management or EGB^29,32,33,39,46,47^ Limited autonomy among clinicians^47^ Lack of environmental awareness and systems thinking^39,40^ Perceived lack of healthcare professional role in sustainability^46^ Lack of confidence, feedback, or belief in impact^31^ Perceived time constraints and competing pressures^29,32,46,47^ Clinical priorities override sustainability^31^ Established habits^29,31,32^ Perception that recycling is too difficult or time-consuming^29^	Environmental awareness and environmental concern^46,47^ Environmental responsibility and personal/intrinsic motivation^30,33,39,46,47^ Motivation reinforced by visible benefits and belief in marginal gains^31^ Integration of sustainability into nursing care through individual actions^40^ Habit formation from repeated sustainable actions^29,31^ Awareness of environmental impacts^29,31^ Rational and mindful use of materials^40^ Emotional impact of visible waste triggering reflection^29^ Desire to implement recycling and minimize waste^29^
Team	Neglect of certain staff groups in training (eg, general services)^40^ Lack of shared knowledge and inconsistent training^40^ Hierarchical or non-collaborative team cultures^31^ Weak influence of environmental attitudes versus peer norms^46^	Peer feedback and informal correction^40^ Peer collaboration and open dialogue^31,47^ Shared responsibility and professional identity as sustainability advocates^40^
Leadership	Lack of leadership engagement and communication^29,47^	Supportive and visible leadership role models^31,47^ Recognition that leadership can shift attitudes and norms^29^ Leadership-driven policies and education^31^
Organizational	Poor or insufficient infrastructure (eg, poor waste bins, labeling, recycling systems)^29,30,33,40,46^ Fragmented procurement and overreliance on disposables^30^ Material oversupply and waste^30^ Inadequate oversight and feedback mechanisms^40^ Fast-paced settings and heavy workload^32,33,47^ Cultural resistance or dismissal of environmental concerns and prioritization of other values (eg, patient safety)^29,31,32,47^ Lack of policy alignment or systemic support^32,46,47^ Absence of institutional ownership or strategy^29^ Inadequate initial or ongoing education^33^	Clear and visible infrastructure (eg, labeled bins, recycling availability)^30,39,40^ Infrastructure promoting environmental cues and nudges (eg, reminders, placement)^31^ Environmental restructuring and conscious design of materials and flows^30^ Institutional oversight through audits, incentives, and monitoring^30,40,46^ Supportive policy, industry collaboration, and regulatory incentives^46,47^ Early and continuing education promoting sustainable practices^29,33,47^ Procurement favoring reusable and high-quality materials^30^

Abbreviation: EGB, employee green behavior.

####  Individual-Level Barriers and Facilitators

 Barriers at the individual level included limited or fragmented knowledge regarding how to execute waste management or EGB,^29,32,33,39,46,47^ a lack of autonomy among clinicians,^47^ and insufficient environmental awareness or systems thinking.^39,40^ Individual perception that sustainability was beyond clinical responsibilities^46^ and doubts about the effectiveness of individual actions^31^ were also identified as barriers. Additional barriers included perceived time constraints,^29,32,46,47^ competing clinical demands,^31^ and ingrained habits that impeded the adoption of sustainable practices.^29,31,32^

 Individual-level facilitators included environmental awareness and personal responsibility toward sustainability.^33,39,46,47^ Intrinsic motivations, such as concern for future generations and emotional reactions to visible waste,^30,33,47^ were also important for encouraging EGB. One study reported that when individuals perceived tangible environmental benefits or believed that small actions could lead to meaningful change, their motivation to act sustainably was strengthened.^31^ In some cases, EGB became embedded in daily practice when environmental responsibility was seen as part of one’s professional role and when deliberate, repeated actions helped form sustainable habits.^29,31,40^

####  Team-Level Barriers and Facilitators

 Team-level barriers included inconsistent training, lack of shared knowledge among team members, and the exclusion of specific staff groups, such as general service workers, from sustainability training.^40^ Non-collaborative or hierarchical team cultures were also identified as barriers.^31^ One study noted that peer norms sometimes conflicted with individual environmental attitudes, limiting behavioral alignment with sustainability values.^46^

 Facilitators included peer feedback and informal correction,^40^ peer collaboration, and open communication.^31,47^ A sense of shared responsibility and professional identity as sustainability advocates was also reported to encourage pro-environmental behavior.^40^

####  Leadership-Level Barriers and Facilitators

 Leadership-level barriers included a lack of engagement and communication regarding sustainable practices.^29,47^ In contrast, facilitators included supportive and visible leadership role models,^31,47^ with some studies recognizing that leadership can shift attitudes and norms towards sustainability.^29^ Additional facilitators included leadership actions such as leadership-driven education and policies.^31^

####  Organizational-Level Barriers and Facilitators 

 Organizational-level barriers included infrastructure-related challenges such as poorly placed or inadequately maintained recycling bins.^29,30,33,40,46^ Several operational and systemic issues were also identified, including fragmented procurement processes,^30^ overreliance on disposable products, and oversupply of materials leading to unnecessary waste,^30^ and inadequate oversight and feedback mechanisms.^40^ Additionally, heavy workloads and the fast-paced nature of clinical settings,^32,33,47^ as well as cultural resistance to change,^31,32,47^ were identified as barriers. Other barriers included a lack of policy alignment or systemic support,^32,46,47^ absence of institutional ownership or strategic direction,^29^ and inadequate initial or ongoing education.^33^

 Facilitators included the implementation of practical infrastructure, such as clearly labeled bins and readily available recycling options.^30,39,40^ Environmental cues and nudges, such as reminders and the strategic placement of bins, were also reported as supportive,^31^ along with efforts to restructure environments through the intentional design of materials and workflow.^30^ Additional facilitators included regulatory audits and policy-based mechanisms such as incentives, monitoring, and enforcement.^30,40,46,47^ Finally, early and continuing education on sustainable practices was also identified as a facilitator.^29,33,47^

## Discussion

 Overall Findings

 This scoping review reflects a growing body of research on EGB in hospital settings, while simultaneously revealing limitations that constrain the development of context-specific and theoretically robust knowledge. Although the included studies were conducted across multiple regions, research activity was concentrated in a small number of countries, with several studies appearing to draw on overlapping institutional samples. In addition, many studies did not report participants’ occupational roles in sufficient detail,^8,42-45^ limiting insight into how different professional groups engage with EGB. This lack of occupational specificity restricts cross-role comparison and may obscure important differences in behavioral opportunities and constraints within hospitals.

 Methodologically, most studies relied on cross-sectional survey designs, which restrict causal interpretation and limit insight into how EGB develops or changes over time. Although some studies drew on theoretical frameworks, their application varied across studies, and several lacked explicit conceptual grounding. These findings suggest that current evidence remains fragmented and underscore the need for more rigorous, theoretically grounded, and contextually sensitive research on EGB in hospitals.

###  Conceptual Inconsistencies in Hospital-Based Green Behavior Research

 A key issue identified in this review is the lack of conceptual consistency in how EGB is defined and operationalized in hospital-based studies. Terms such as “green behavior,” “pro-environmental behavior,” and “EGB” were often used interchangeably, yet referred to different constructs ranging from discretionary workplace actions^42^ to environmentally oriented behavior^9^ and specific practices such as energy conservation.^45^ In addition, several studies used the same measurement scales despite differing underlying conceptual assumptions, which raises questions about construct alignment and comparability. These inconsistencies complicate the synthesis of findings across studies and limit cumulative theory development. Without clearer conceptual boundaries, it remains difficult to determine whether studies are examining comparable constructs or context-specific forms of EGB within hospitals.

###  Proposed Working Definition of Employee Green Behavior

 In light of the conceptual and operational inconsistencies identified in the reviewed studies, a clear and context-sensitive working definition of EGB is needed for hospital-based research. Based on the synthesis of existing definitions, we propose the following working definition: *EGB refers to actions undertaken by employees in the workplace to minimize environmental harm and promote sustainability. These behaviors may extend beyond formal job roles and contribute to organizational and societal environmental goals. *In hospital settings, these behaviors are often enacted within the constraints of clinical workflows, patient safety requirements, and organizational routines. This definition is intended as a working, rather than definitive, formulation. It does not assume that motivation, voluntariness, or impact are uniform across different hospital roles and contexts, and it remains sensitive to the professional norms and operational realities of healthcare work. Future empirical research is needed to refine, test, and contextualize this definition across different occupational groups and healthcare systems.

###  Factors Associated with Employee Green Behavior Across Hospital Contexts

 Integrating quantitative and qualitative findings suggests that EGB in hospitals is associated with factors spanning multiple levels of the organizational context, including individual, team, leadership, and organizational levels. At the individual level, studies reported associations between EGB and factors such as environmental responsibility rooted in professional identity^39^ or values such as altruism.^8,9^ However, individual motivation alone may be insufficient to explain engagement in EGB, particularly in the presence of clinical demands and time constraints. Structural conditions such as high patient acuity, heavy workload, and rapid staff turnover^31,33,47^ were described as contextual features associated with reduced opportunities to enact EGB.

 At the team level, factors such as knowledge sharing,^41^ and peer feedback and collaboration^40^ wereassociated with EGB, whereas hierarchical or non-supportive team cultures were described as barriers.^31^ At the leadership level, supportive leadership practices^8,28,29,31,41,43-45,47^ were frequently discussed in relation to higher levels of EGB across studies.

 At the organizational level, studies described structural and cultural features linked to EGB, including access to recycling infrastructure, availability of training, organizational sustainability orientation, and perceived organizational support.^30,46^ These factors were typically discussed as contextual conditions influencing the likelihood of EGB rather than as direct causal determinants. These findings highlight the multilevel nature of factors associated with EGB in hospital settings.

###  Theoretical Interpretation in Hospital Context

 Although this scoping review did not aim to test or extend specific organizational theories, the findings can be interpreted through a job demands-resources perspective. Across studies, workload pressures, time constraints, and limited resources were frequently described as barriers to EGB.^32,47^ From a job demands-resources perspective,^56^ these conditions can be understood as job demands that may limit employees’ capacity to engage in discretionary or extra-role behaviors, including environmentally responsible practices. In contrast, organizational support such as leadership encouragement,^47^ training opportunities,^33^ access to recycling infrastructure,^30^ and opportunities for knowledge sharing^41^ may function as job resources that enable or facilitate green behavior in the workplace. In this sense, many of the factors identified in this review can be interpreted as reflecting the balance between job demands and job resources within hospital work environments. Interpreted through this lens, the findings suggest that EGB in hospital settings is shaped not only by individual motivation but also by the broader configuration of demands and resources embedded in clinical work settings.

###  Positioning Hospital Findings Within Existing Literature

 The findings identified in this review broadly correspond with existing organizational research that conceptualizes EGB as embedded within organizational contexts.^13^ However, hospital settings present distinctive characteristics that differentiate them from other sectors. For example, fast-paced workflows, high patient acuity, and frequent staff turnover were repeatedly described as contextual features that may challenge consistent engagement in EGB.^31,33,47^ Hospitals also function as training environments with rotating students and temporary staff, which may disrupt continuity in sustainability practices.^33^ These contextual characteristics suggest that findings from non-healthcare sectors cannot be directly transferred to hospital settings without careful consideration. By focusing specifically on hospital-based studies, this review extends prior sustainability research in healthcare^57^ by synthesizing how EGB has been conceptualized, studied, and supported within hospital contexts.

###  Integrative Synthesis of Reported Factors

Figure 2 provides a conceptual overview of factors associated with EGB identified across the included studies and organizes them across four levels: individual, team, leadership, and organization. This categorization is intended to clarify the scope of existing research rather than to represent empirically tested pathways or interactions.

**Figure 2:**
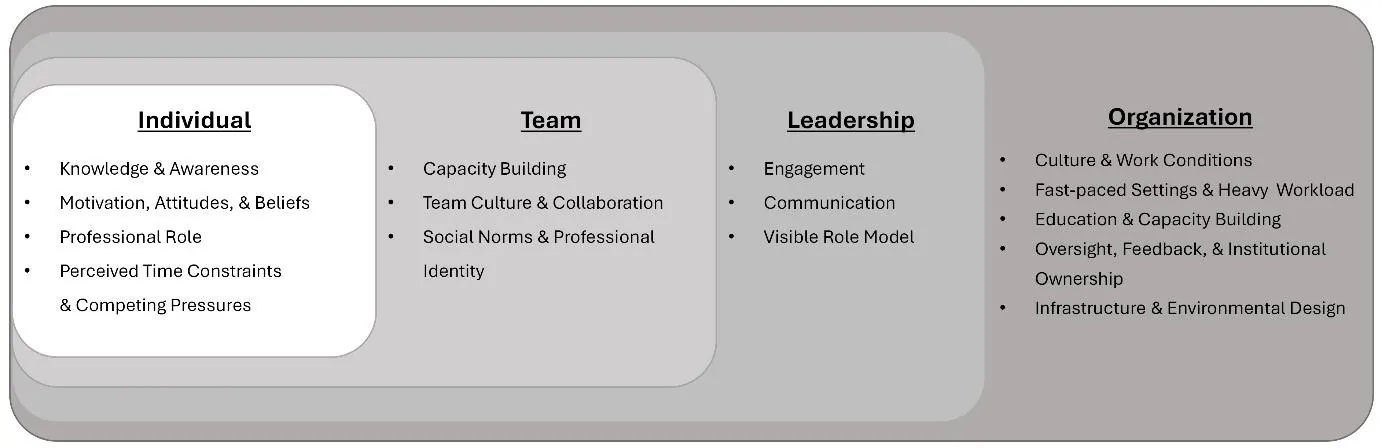
Conceptual Overview of Factors Associated with Employee Green Behavior Reported in Hospital-Based Studies. The figure summarizes factors identified across the reviewed literature and organizes them across four levels: individual, team, leadership, and organization. This visualization represents a synthesis of reported factors rather than an empirically tested model and does not depict directional or causal relationships.

 Across studies, individual motivation was frequently discussed alongside contextual conditions related to teams, leadership, and organizational structures. For example, studies described situations in which environmentally motivated staff were unable to act due to time pressures or inadequate infrastructure,^28,40,47^ as well as cases in which organizational initiatives showed limited uptake when they were not aligned with professional routines or values.^9,42^ Leadership was commonly described as a contextual factor that helps translate organizational sustainability priorities into day-to-day practices.^31,41,45^ These observations highlight how EGB in hospital settings appears to be shaped by both individual motivation and the surrounding clinical and organizational context.

###  Future Directions and Implications 

####  Research

 Future research should aim to improve conceptual clarity through the adoption of consistent definitions of EGB. In addition, longitudinal and mixed-method designs may help advance understanding of how EGB evolves over time and how it differs across professional groups within hospital settings.

####  Employee Education

 Across studies, knowledge, awareness, education, and capacity building were consistently reported as both barriers to and facilitators of EGB.^29,31-33,39,40,46,47^ Integrating sustainability into initial professional education and reinforcing it through ongoing training may support engagement in environmentally responsible practices.^29,33,47^ These findings suggest that sustainability education should be embedded across all stages of health professional education and continuing professional development.

####  Practice

 Promoting EGB in hospitals requires attention to organizational context in addition to individual motivation. While interventions such as training programs^35^ and institutional policies^8^ were frequently described, their reported effectiveness varied depending on clinical demands, organizational structures, and leadership practices.^31^ Hospitals may therefore benefit from aligning sustainability initiatives with routine workflows and professional priorities to support feasible and context-sensitive implementation.

####  Policy

 Some studies highlighted the importance of oversight mechanisms, feedback systems, and institutional alignment in supporting sustainability initiatives.^46,47^ Coordinated policy approaches at organizational and system levels may strengthen support for EGB across healthcare systems, particularly when sustainability objectives extend beyond direct clinical care.

###  Limitations

 Several limitations of this review should be noted. First, the included studies were heterogeneous in terms of study designs, theoretical framing, and measurement approaches. In addition, the predominance of cross-sectional, self-reported survey data limits causal interpretation and raises the possibility of common method bias, thereby constraining the strength of the conclusions drawn from the synthesized findings. Second, although the studies covered multiple regions, research activity was concentrated in a small number of countries and institutions. Several studies appeared to draw on overlapping samples, particularly within specific national contexts, which may have contributed to the over-representation of certain findings. This concentration may limit the transferability of insights to other healthcare systems and organizational contexts. Third, only English-language publications were included, which may have introduced language bias. While the search strategy was comprehensive and included selected grey literature sources such as theses, this review focused on empirical research and therefore excluded non-research materials (eg, policy reports or organizational documents). As a result, some practice- or policy-oriented perspectives may not be fully represented. Finally, consistent with scoping review methodology,^19^ no formal critical appraisal of study quality was conducted. Consequently, the synthesis does not differentiate findings based on the methodological rigor of individual studies.

## Conclusion

 This scoping review examined how EGB has been conceptualized and studied within hospital settings. Although scholarly interest in this topic has increased in recent years, the findings reveal considerable variability in terminology, definitions, and measurement approaches, which limits the development of cumulative knowledge and comparison across studies. To address this issue, this review proposed a context-sensitive working definition of EGB to support greater conceptual clarity in future hospital-based research. The review also identified factors related to EGB at the individual, team, leadership, and organizational levels, highlighting the multilevel nature of environmentally responsible behavior in hospital organizations. These factors should not be interpreted as causal determinants but as patterns reported across studies within the operational realities of healthcare environments, including heavy workloads, hierarchical decision-making, and dynamic team structures. Future research should prioritize clearer conceptualization, more consistent theoretical framing, and study designs that are better capture context-specific patterns of EGB within clinical settings. Greater attention to professional roles, team dynamics, and institutional culture may support the development of more feasible and sustainable approaches to promoting environmental responsibility in hospital settings.

## Acknowledgements

 The authors thank Dr. Ja Kyung Seo for her support with literature search.

## Disclosure of artificial intelligence (AI) use

 ChatGPT was used solely for language editing and improvement of readability.

## Ethical issues

 Not applicable.

## Conflicts of interest

 Authors declare that they have no conflicts of interest.

## Disclaimer

 The opinions expressed herein are solely those of the authors.

## Supplementary files


Supplementary file 1 contains Table S1.

Supplementary file 2 contains Figure S1.
