# Supplementary material for: Employee Green Behavior in Hospitals: A Scoping Review of Definitions and Influencing Factors
Source: Int J Health Policy Manag. 2026 Jul 14;15:9377. doi: 10.34172/ijhpm.9377 (PMC13770231; doi:10.34172/ijhpm.9377)
Supplement: Supplementary file 1 [file ijhpm-15-9377-s001.pdf]

**Article title:** Employee Green Behavior in Hospitals: A Scoping Review of Definitions and Influencing Factors

**Journal name:** International Journal of Health Policy and Management (IJHPM)

**Authors' information**: Seung Eun Lee[1]*, Heejin Lim[1], Hyeyeon Shin[2], Sungwon Park[3], Jin Jun[2]

[1]College of Nursing, Yonsei University, Seoul, Republic of Korea.

[2]College of Nursing, Ohio State University, Columbus, OH, USA.

[3]School of Nursing, University of California, Los Angeles, CA, USA.

***Correspondence to:** Seung Eun Lee; Email: leese@yuhs.ac

**Citation:** Lee SE, Lim H, Shin H, Park S, Jun J. Employee green behavior in hospitals: a scoping review of definitions and influencing factors. Int J Health Policy Manag. 2026;15:9377. doi:10.34172/ijhpm.9377

**Supplementary file 1**

**Table S1.** Search Strategy

| Database | | Search terms |
|---|---|---|
| PubMed -Result: 1,094 | #1 | ((((Employee*[Title/Abstract]) OR (Individual*[Title/Abstract])) OR (Worker*[Title/Abstract])) OR (personnel[Title/Abstract])) OR ("Health Personnel"[Mesh]) |
| | #2 | (((Hospital[Mesh]) OR (Health Facilities[Mesh])) OR (Healthcare*[Title/Abstract])) OR ("Healthcare setting*"[Title/Abstract]) |
| | #3 | (((((((((((((((((((("Green behavio*"[Title/Abstract]) OR ("Environment friendly behavio*"[Title/Abstract])) OR ("Environmental friendly behavio*"[Title/Abstract])) OR ("Environment-friendly behavio*"[Title/Abstract])) OR ("Environmental-friendly behavio*"[Title/Abstract])) OR ("Environmentally friendly behavio*"[Title/Abstract])) OR ("Environmentally-friendly behavio*"[Title/Abstract])) OR (Pro-environment*[Title/Abstract] AND behavio*[Title/Abstract])) OR (Proenvironment*[Title/Abstract] AND behavio*[Title/Abstract])) OR ("Sustainable |

| | | |
|---|---|---|
| | | Behavio*"[Title/Abstract])) OR (Eco[Title/Abstract] AND behavio*[Title/Abstract])) OR (ecofriend*[Title/Abstract] AND behavio*[Title/Abstract])) OR ("Environmental Protection"[Title/Abstract])) OR ("Waste Management"[Mesh])) OR ("Recycling"[Mesh])) OR (recycl*[Title/Abstract])) OR (reuse[Title/Abstract])) OR (re-use[Title/Abstract])) OR (environment*[Title/Abstract] AND "Organizational Citizenship Behavio*"[Title/Abstract])) OR (green[Title/Abstract] AND "Organizational Citizenship Behavio*"[Title/Abstract])) OR (environment*[Title/Abstract] AND "prosocial behavio*"[Title/Abstract])) OR (green[Title/Abstract] AND "prosocial behavio*"[Title/Abstract]) |
| | #4 | #1 AND #2 AND #3 |
| CINAHL<br>-Result: 252 | #1 | ( TI Employee* OR TI Individual* OR TI Worker* OR TI personnel OR TI "Health Personnel" OR AB Employee* OR AB Individual* OR AB Worker* OR AB personnel OR AB "Health Personnel") |
| | #2 | (TI Hospital* OR TI "Health Facilit*" OR TI Healthcare* OR TI "Healthcare setting*" OR AB Hospital* OR AB "Health Facilit*" OR AB Healthcare* OR AB "Healthcare setting*") |
| | #3 | TI “Green behavio*” OR AB “Green behavio*” OR TI “Environment friendly behavio*” OR AB “Environment friendly behavio*” OR TI “Environmental friendly behavio*” OR AB “Environmental friendly behavio*” OR TI “Environment-friendly behavio*” OR AB “Environment-friendly behavio*” OR TI “Environmental-friendly behavio*” OR AB “Environmental-friendly behavio*” OR TI “Environmentally friendly behavio*” OR AB “Environmentally friendly behavio*” |
| | #4 | TI “Environmentally-friendly behavio*” OR AB “Environmentally-friendly behavio*” OR TI ( Pro-environment* AND behavio* ) OR AB ( Pro-environment* AND behavio* ) OR TI ( Proenvironment* AND behavio* ) OR AB ( Proenvironment* AND behavio* ) OR TI |

| | | |
|---|---|---|
| | | “Sustainable Behavio*” OR AB “Sustainable Behavio*” OR TI ( Eco AND behavio* ) OR AB ( Eco AND behavio* ) OR TI ( ecofriend* AND behavio* ) OR AB ( ecofriend* AND behavio* ) |
| | #5 | TI “Environmental Protection” OR AB “Environmental Protection” OR TI “Waste Management” OR AB “Waste Management” OR TI recycl* OR AB recycl* OR TI reuse OR AB reuse OR TI re-use OR AB re-use OR TI ( environment* AND "Organizational Citizenship Behavio*" ) OR AB ( environment* AND "Organizational Citizenship Behavio*" ) |
| | #6 | TI ( green AND "Organizational Citizenship Behavio*" ) OR AB ( green AND "Organizational Citizenship Behavio*" ) OR TI ( environment* AND "prosocial behavio*" ) OR AB ( environment* AND "prosocial behavio*" ) OR TI ( green AND "prosocial behavio*" ) OR AB ( green AND "prosocial behavio*" ) |
| | #7 | #3 OR #4 OR #5 OR #6 |
| | #8 | #1 AND #2 AND #7 |
| Embase -Result: 1,050 | #1 | employee*:ab,ti OR individual*:ab,ti OR worker*:ab,ti OR personnel:ab,ti OR 'health personnel':ab,ti |
| | #2 | hospital*:ab,ti OR 'health facilit*':ab,ti OR healthcare*:ab,ti OR 'healthcare setting*':ab,ti |
| | #3 | 'green behavio*':ab,ti OR 'environment friendly behavio*':ab,ti OR 'environmental friendly behavio*':ab,ti OR 'environment-friendly behavio*':ab,ti OR 'environmental-friendly behavio*':ab,ti OR 'environmentally friendly behavio*':ab,ti OR 'environmentally-friendly behavio*':ab,ti OR ('pro-environment*':ab,ti AND behavio*:ab,ti) OR (proenvironment*:ab,ti AND behavio*:ab,ti) OR 'sustainable behavio*':ab,ti OR (eco:ab,ti AND behavio*:ab,ti) OR (ecofriend*:ab,ti AND behavio*:ab,ti) OR 'environmental protection':ab,ti OR 'waste management':ab,ti OR recyl*:ab,ti OR reuse:ab,ti OR 're-use':ab,ti OR (environment*:ab,ti AND 'Organizational Citizenship Behavio*':ab,ti) OR (green:ab,ti AND |

| | | |
|---|---|---|
| | | 'Organizational Citizenship Behavio*':ab,ti) OR (environment*:ab,ti AND 'prosocial Behavio*':ab,ti) OR (green:ab,ti AND 'prosocial Behavio*':ab,ti) |
| | #4 | #1 AND #2 AND #3 |
| Web of Science -Result: 1,106 | #1 | TI=(Employee*) OR AB=(Employee*) OR TI=(Individual*) OR AB=(Individual*) OR TI=(Worker*) OR AB=(Worker*) OR TI=(personnel) OR AB=(personnel) OR TI=("Health Personnel") OR AB=("Health Personnel") |
| | #2 | TI=(Hospital*) OR AB=(Hospital*) OR TI=("Health Facilit*") OR AB=("Health Facilit*") OR TI=(Healthcare*) OR AB=(Healthcare*) OR TI=("Healthcare setting*") OR AB=("Healthcare setting*") |
| | #3 | TI=(“Green behavio*”) OR AB=(“Green behavio*”) OR TI=(“Environment friendly behavio*”) OR AB=(“Environment friendly behavio*”) OR TI=(“Environmental friendly behavio*”) OR AB=(“Environmental friendly behavio*”) OR TI=(“Environment-friendly behavio*”) OR AB=(“Environment-friendly behavio*”) OR TI=(“Environmental-friendly behavio*”) OR AB=(“Environmental-friendly behavio*”) OR TI=(“Environmentally friendly behavio*”) OR AB=(“Environmentally friendly behavio*”) OR TI=(“Environmentally-friendly behavio*”) OR AB=(“Environmentally-friendly behavio*”) OR TI=(Pro-environment* AND behavio*) OR AB=(Pro-environment* AND behavio*) OR TI=(Proenvironment* AND behavio*) OR AB=(Proenvironment* AND behavio*) OR TI=(“Sustainable Behavio*” ) OR AB=(“Sustainable Behavio*” ) OR TI=(Eco AND behavio*) OR AB=(Eco AND behavio*) OR TI=(ecofriend* AND behavio*) OR AB=(ecofriend* AND behavio*) OR TI=(“Environmental Protection” ) OR AB=(“Environmental Protection” ) OR TI=("Waste Management") OR AB=("Waste Management") OR TI=(recycl*) OR AB=(recycl*) OR TI=(reuse) OR AB=(reuse) OR TI=(re-use) OR AB=(re-use) OR TI=(environment* |

| | | |
|---|---|---|
| | | AND "Organizational Citizenship Behavio*") OR AB=(environment* AND "Organizational Citizenship Behavio*") OR TI=(green AND "Organizational Citizenship Behavio*") OR AB=(green AND "Organizational Citizenship Behavio*") OR TI=(environment* AND "prosocial Behavio*") OR AB=(environment* AND "prosocial Behavio*") OR TI=(green AND "prosocial Behavio*") OR AB=(green AND "prosocial Behavio*") |
| | #4 | #1 AND #2 AND #3 |
| Scopus -Result: 1,948 | #1 | TITLE-ABS(Employee* OR Individual* OR Worker* OR personnel OR "Health Personnel") |
| | #2 | TITLE-ABS(Hospital* OR "Health Facilit*" OR Healthcare* OR "Healthcare setting*") |
| | #3 | TITLE-ABS("Green behavio*") OR TITLE-ABS("Environment friendly behavio*") OR TITLE-ABS("Environmental friendly behavio*") OR TITLE-ABS("Environment-friendly behavio*") OR TITLE-ABS("Environmental-friendly behavio*") OR TITLE-ABS("Environmentally friendly behavio*") OR TITLE-ABS("Environmentally-friendly behavio*") OR TITLE-ABS(Pro-environment* AND behavio*) OR TITLE-ABS(Proenvironment* AND behavio*) OR TITLE-ABS("Sustainable Behavio*" ) OR TITLE-ABS(Eco AND behavio*) OR TITLE-ABS(ecofriend* AND behavio*) OR TITLE-ABS("Environmental Protection" ) OR TITLE-ABS(Waste Management) OR TITLE-ABS(recycl*) OR TITLE-ABS(reuse) OR TITLE-ABS(re-use) OR TITLE-ABS(environment* AND "Organizational Citizenship Behavio*") OR TITLE-ABS(green AND "Organizational Citizenship Behavio*") OR TITLE-ABS(environment* AND "prosocial Behavio*") OR TITLE-ABS(green AND "prosocial Behavio*") |
| | #4 | #1 AND #2 AND #3 |
| PsycINFO -Result: 106 | #1 | tiab(Employee*) OR tiab(Individual*) OR tiab(Worker*) OR tiab(personnel) OR tiab("health personnel") |

| | | |
|---|---|---|
| | #2 | tiab(Hospital*) OR tiab("Health Facilit*") OR tiab("Healthcare setting*") OR tiab(Healthcare*) |
| | #3 | tiab(“Green behavio*”) OR tiab(“Environment friendly behavio*”) OR tiab(“Environmental friendly behavio*”) OR tiab(“Environment-friendly behavio*”) OR tiab(“Environmental-friendly behavio*”) OR tiab(“Environmentally friendly behavio*”) OR tiab(“Environmentally-friendly behavio*”) OR tiab(Pro-environment* AND behavio*) OR tiab(Proenvironment* AND behavio*) OR tiab(“Sustainable Behavio*”) |
| | #4 | tiab(Eco AND behavio*) OR tiab(ecofriend* AND behavio*) OR tiab(“Environmental Protection” ) OR tiab(Waste Management) OR tiab(recycl*) OR tiab(reuse) OR tiab(re-use) OR tiab(environment* AND "Organizational Citizenship Behavio*") OR tiab(green AND "Organizational Citizenship Behavio*") |
| | #5 | tiab(environment* AND "prosocial Behavio*") OR tiab(green AND "prosocial Behavio*") |
| | #6 | #3 OR #4 OR #5 |
| | #7 | #1 AND #2 AND #6 |
| Emerald journals -Result: 483 | #1 | (title:"Employee*") OR (title:"Individual*") OR (title:"Worker*") OR (title:"personnel") OR (title:"Health Personnel") OR (abstract:"Individual*") OR (abstract:"Worker*") OR (abstract:"personnel") OR (abstract:"Employee*") OR (abstract:"Health Personnel") |
| | #2 | (title:"Hospital*") OR (abstract:"Hospital*") OR (title:"Health Facilit*") OR (abstract:"Health Facilit*") OR (title:"Healthcare*") OR (abstract:"Healthcare*") OR (title:"Healthcare setting*") OR (abstract:"Healthcare setting*") |
| | #3 | (title:“Green behavio*”) OR (abstract:“Green behavio*”) OR (title:“Environment friendly behavio*”) OR (abstract:“Environment friendly behavio*”) OR (title:“Environmental friendly behavio*”) OR (abstract:“Environmental friendly behavio*”) OR (title:“Environment- |

| | | |
|---|---|---|
| | | friendly behavio*”) OR (abstract:“Environment-friendly behavio*”) OR (title:“Environmental-friendly behavio*”) OR (abstract:“Environmental-friendly behavio*”) OR (title:“Environmentally friendly behavio*”) OR (abstract:“Environmentally friendly behavio*”) OR (title:“Environmentally-friendly behavio*”) OR (abstract:“Environmentally-friendly behavio*”) OR (title:Pro-environment* AND behavio*) OR (abstract:Pro-environment* AND behavio*) OR (title:Proenvironment* AND behavio*) OR (abstract:Proenvironment* AND behavio*) OR (title:“Sustainable Behavio*” ) OR (abstract:“Sustainable Behavio*” ) OR (title:Eco AND behavio*) OR (abstract:Eco AND behavio*) OR (title:ecofriend* AND behavio*) OR (abstract:ecofriend* AND behavio*) OR (title:“Environmental Protection” ) OR (abstract:“Environmental Protection” ) OR (title:“Waste Management”) OR (abstract:“Waste Management”) OR (title:recycl*) OR (abstract:recycl*) OR (title:reuse) OR (abstract:reuse) OR (title:re-use) OR (abstract:re-use) OR (title:environment* AND "Organizational Citizenship Behavio*") OR (abstract:environment* AND "Organizational Citizenship Behavio*") OR (title:green AND "Organizational Citizenship Behavio*") OR (abstract:green AND "Organizational Citizenship Behavio*") OR (title:environment* AND "prosocial Behavio*") OR (abstract:environment* AND "prosocial Behavio*") OR (title:green AND "prosocial Behavio*") OR (abstract:green AND "prosocial Behavio*") |
| | #4 | #1 AND #2 AND #3 |
| ProQuest Dissertations & Theses Global -Result: 305 | #1 | tiab(Employee*) OR tiab(Individual*) OR tiab(Worker*) OR tiab(personnel) OR tiab("health personnel") |
| | #2 | tiab(Hospital*) OR tiab("Health Facilit*") OR tiab("Healthcare setting*") OR tiab(Healthcare*) |
| | #3 | tiab(“Green behavio*”) OR tiab(“Environment friendly behavio*”) OR |

| | |
|---|---|
| | tiab(“Environmental friendly behavio*”) OR tiab(“Environment-friendly behavio*”) OR tiab(“Environmental-friendly behavio*”) OR tiab(“Environmentally friendly behavio*”) OR tiab(“Environmentally-friendly behavio*”) OR tiab(Pro-environment* AND behavio*) OR tiab(Proenvironment* AND behavio*) OR tiab(“Sustainable Behavio*”) |
| #4 | tiab(Eco AND behavio*) OR tiab(ecofriend* AND behavio*) OR tiab(“Environmental Protection” ) OR tiab(Waste Management) OR tiab(recycl*) OR tiab(reuse) OR tiab(re-use) OR tiab(environment* AND "Organizational Citizenship Behavio*") OR tiab(green AND "Organizational Citizenship Behavio*") |
| #5 | tiab(environment* AND "prosocial Behavio*") OR tiab(green AND "prosocial Behavio*") |
| #6 | #3 OR #4 OR #5 |
| #7 | #1 AND #2 AND #6 |
