# Supplementary material for: Employee Green Behavior in Hospitals: A Scoping Review of Definitions and Influencing Factors
Source: Int J Health Policy Manag. 2026 Jul 14;15:9377. doi: 10.34172/ijhpm.9377 (PMC13770231; doi:10.34172/ijhpm.9377)
Supplement: Supplementary file 2 [file ijhpm-15-9377-s002.pdf]

Article title: Employee Green Behavior in Hospitals: A Scoping Review of Definitions and Influencing Factors

Journal name: International Journal of Health Policy and Management (IJHPM)

Authors' information: Seung Eun Lee[1]*, Heejin Lim[1], Hyeyeon Shin[2], Sungwon Park[3], Jin Jun[2]

[1]College of Nursing, Yonsei University, Seoul, Republic of Korea.

[2]College of Nursing, Ohio State University, Columbus, OH, USA.

[3]School of Nursing, University of California, Los Angeles, CA, USA.

***Correspondence to:** Seung Eun Lee; Email: leese@yuhs.ac

Citation: Lee SE, Lim H, Shin H, Park S, Jun J. Employee green behavior in hospitals: a scoping review of definitions and influencing factors. Int J Health Policy Manag. 2026;15:9377. doi:10.34172/ijhpm.9377

Supplementary file 2

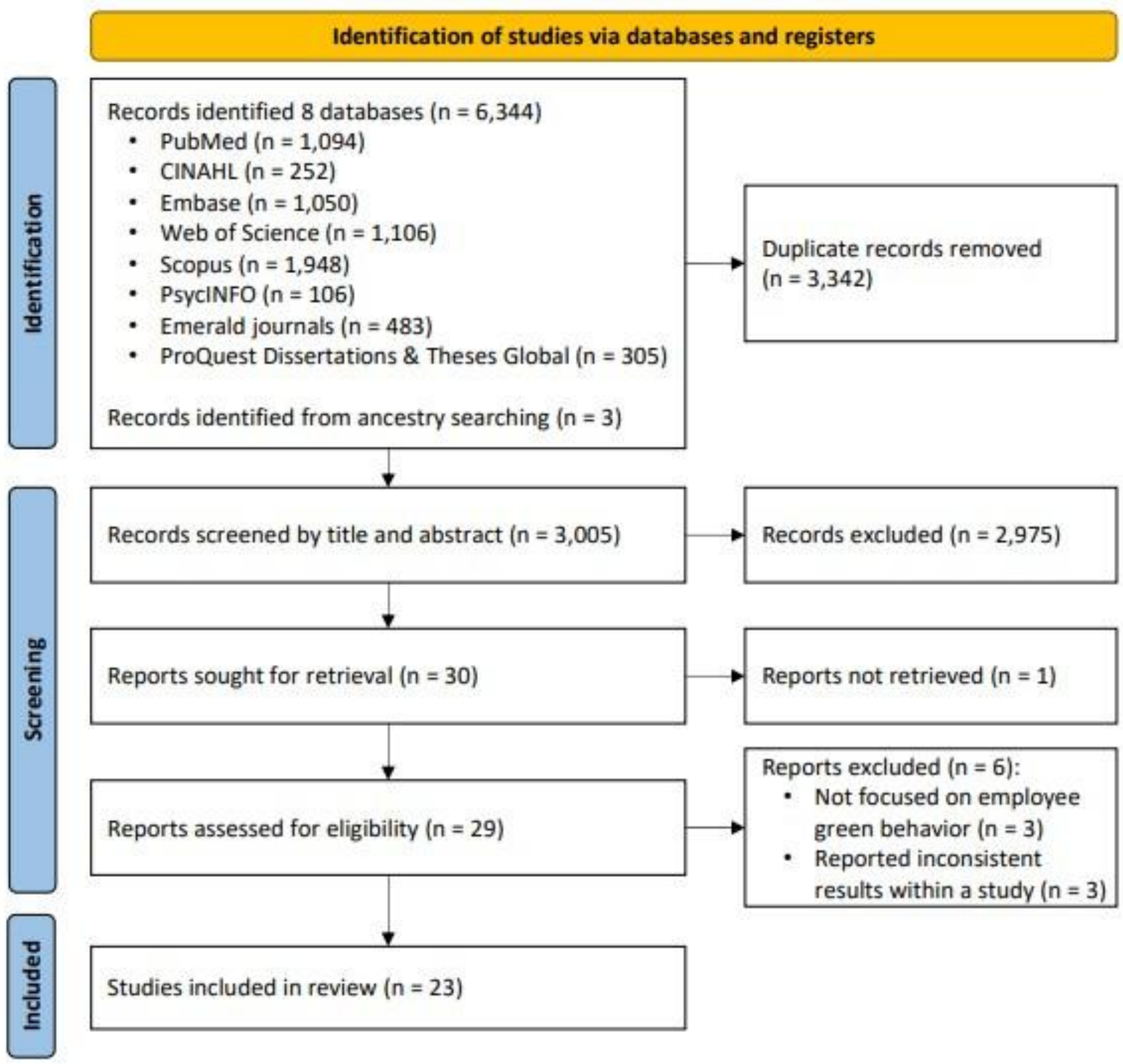

Figure S1. Preferred Reporting Items for Systematic Reviews and Meta-Analysis Extension Flowchart Diagram
